# Mechanical Punctate Pain Thresholds in Patients With Migraine Across Different Migraine Phases: A Narrative Review

**DOI:** 10.3389/fneur.2021.801437

**Published:** 2022-01-28

**Authors:** Li-Ling Hope Pan, Rolf-Detlef Treede, Shuu-Jiun Wang

**Affiliations:** ^1^Brain Research Center, National Yang Ming Chiao Tung University, Taipei, Taiwan; ^2^Chair of Neurophysiology, Mannheim Center for Translational Neurosciences, Medical Faculty Mannheim, Heidelberg University, Mannheim, Germany; ^3^College of Medicine, National Yang Ming Chiao Tung University, Taipei, Taiwan; ^4^Department of Neurology, Neurological Institute, Taipei Veterans General Hospital, Taipei, Taiwan

**Keywords:** pain sensitivity, allodynia, quantitative sensory testings, migraine cycle, fluctuation

## Abstract

**Purpose of the Review:**

We reviewed the studies of mechanical punctate pain thresholds (MPTs) in patients with migraine and summarized their findings focusing on the differences in MPT measurement and MPTs in different phases of migraine.

**Methods:**

We searched the English-written articles that investigate the MPTs in the migraine population published in peer-reviewed journals with full-text using the PubMed, Web of Science, and Google Scholar databases. Moreover, we manually searched the references from the articles for possibly related studies.

**Main Findings:**

We collected 276 articles and finally included twelve studies in this review. Most of the studies that included MPTs were measured with traditional von Frey filaments. The cephalic areas were always included in the assessment. Most studies compared the inter-ictal MPT in patients with migraine to controls. Among them, the majority found no significant differences; however, there were studies found either higher or lower levels of MPTs in migraine. Even though the studies provided the criteria to define the inter-ictal phase, not all of them followed up with the subjects regarding the next migraine attack. In studies that compared MPT between phases, lower MPTs were found during peri-ictal phases.

**Summary:**

Changes to MPT in migraine patients were inconclusive. The selection of measurement methods as well as properly defined migraine phases should be considered for future studies.

## Introduction

Migraine is a prevalent neurological disease that debilitates 14.4% of the population worldwide (1). Migraine is defined as headache attacks lasting 4–72 h that manifest by unilaterality, pulsating quality, moderate or severe pain intensity, and aggravation by or causing avoidance of routine physical activity. It is characterized with nausea, vomiting, photophobia, and phonophobia. Chronic migraine (CM) is diagnosed while headache occurs on ≥ 15 days per month for more than 3 months, which, on at least 8 of them, has the features of migraine headache. On the other hand, those who do not fulfill the diagnostic criteria of CM are classified as episodic migraine (EM) (2). Migraine is known as a sensory threshold disease (3) that fluctuates across four phases, namely inter-ictal, pre-ictal, ictal, and post-ictal phases. The pre-ictal and post-ictal phases represent the periods 24–72 h before the headache attack starts and after the headache attack ceases, respectively. The ictal phase is when the patient is experiencing a headache, while the inter-ictal phase is the period that falls outside of these three phases (i.e., after the post-ictal phase and before the pre-ictal phase of the next migraine).

Several studies have evaluated physiological changes in patients with migraines across these phases using various techniques, including neuroimaging (4) and electrophysiological modalities (5, 6). Even when no headache is experienced, there is evidence that cortical or functional changes begin earlier during the pre-ictal phase (7–13), and last hours to days after the attack, that is, the post-ictal phase (3, 10, 12). For example, the resting EEG power spectral density was higher and the coherence was stronger during pre-ictal phase compared to inter-ictal phase (13). The functional MRI study found increased hypothalamic activity triggered by trigeminal nociceptive stimulation within 24 h before the headache onset (12). Changes to sensory thresholds across migraine phases have been primarily evaluated thermally (8, 14–17). The sensory threshold is defined as the weakest stimuli that can be detected, for example as warm or cold, by the subject at least half of the time and the pain threshold is at the intensity when the stimuli become painful. For instance, compared to the inter-ictal phase, heat pain thresholds (HPTs) tend to decrease during the pre-ictal, ictal, and post-ictal phases (8, 11) in patients with migraine. In contrast, cold pain thresholds (CPTs) (14, 18) show inconsistent results. Thermal pain thresholds in patients with migraine have been previously reviewed (19).

The MPT can be objectively measured using von Frey filaments (20). The traditional von Frey filaments was a set of seven custom-made weighted pinprick stimulators that exert forces between 8 and 512 mN (20). The measurements were done with the “method of limits,” which the assessor searched ascendingly and descendingly for the thresholds. The results of this measurement are nominal and geometric means and logarithmic transformation are recommended for further comparison (20). The electronic von Frey filaments measure the reaction force against the targeted area continuously with a load cell. The results of measurements using electronic von Frey filaments are numeric and can be compared directly (21).

In this review, we aimed to review and summarize the current studies regarding MPT in patients with migraine, including the methodology of MPT measurement and the patient characteristics.

## Methods

We searched the English-written articles that investigate the MPTs in the migraine population published in peer-reviewed journals with full-text using the PubMed, Web of Science, and Google Scholar databases. The search was performed on October 1^st^, 2021, using the keywords of “mechanical pain” and “migraine.” We also searched the references of the articles for possibly related studies. The systematic review/meta-analysis, pre-clinical studies, case reports, the studies not done in adult patients with migraine, and not reported MPT results were excluded from this review.

## Results

**Figure 1:**
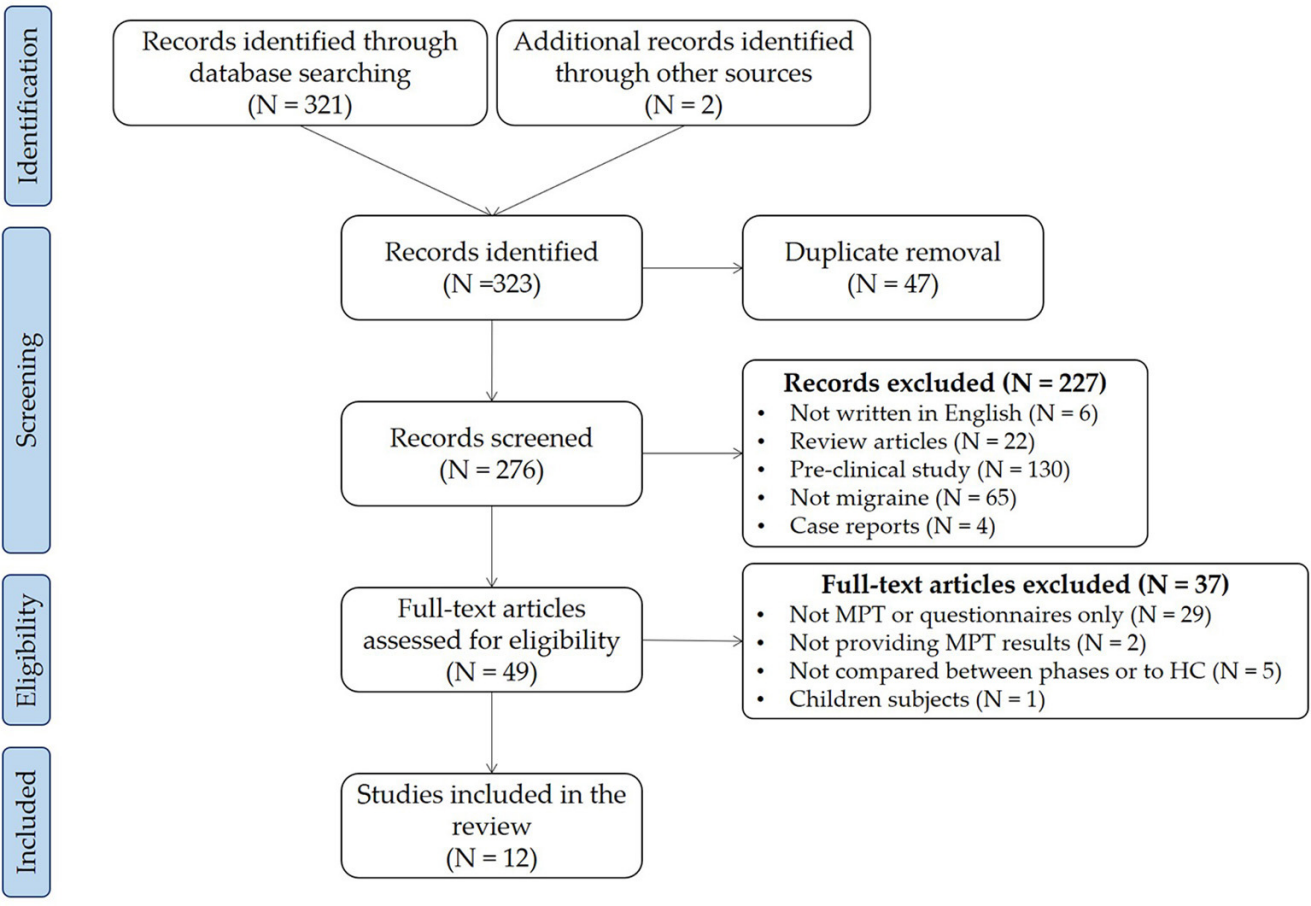
The PRISMA flow diagram.

We collected 274 articles, including six reviews and 268 original articles. In addition, we added two articles manually from the reference of included articles. Finally, we included twelve articles in this review. Figure 1 shows the PRISMA flow chart of the search and Table 1 shows the studies included in the review.

**Table 1:** Studies included in the review.

**References**	**Equipment**	**Subject**	**Location**	**Targeted side**	**Phase**	**Follow-up**	**Main findings**
Burstein et al. (26)	TvFF	42 EM (6 males)	periorbital, ventral forearm	bil.	ictal, migraine-free (7 days since last attack)	no	ictal < migraine-free -allodynic attacks: all sites -non-allodynic attacks: ipsilateral periorbital area only
Weissman-Fogel et al. (17)	TvFF	34 EM (6 males) 28 HC (5 males)	supra-orbital area	painful side	headache-free (7 days since last attack)	no	EM < HC
Yarnitsky et al. (24)	TvFF	23 EM (sex ratio of subgroup was not reported)	forehead, forearm	bil.	inter-ictal, ictal	no	attack < interictal (bil. forehead)
Burstein et al. (27)	TvFF	31 EM (sex ratio not reported)	periorbital	the side of the referred pain	ictal, migraine-free (5 days since last attack)	no	ictal < migraine-free (allodynic migraine attacks)
Schwedt et al. (16)	TvFF	20 CM (2 males) 20 EM (5 males) 20 HC (4 males)	forehead, ventral forearm	bil.	inter-ictal (48 h since last attack)	no	n.s.
Zappaterra et al. (22)	TvFF	27 TM-MOH (8 males) 21 EM (4 males) 26 HC (9 males)	temple, cheekbone, neck	bil.	headache-free	no	TM-MOH < EM < HC
Beese et al. (18)	TvFF	22 EM (0 male) 22 HC (0 male)	cheek, ventral forearm	bil.	inter-ictal (48 h since last attack, 48 h before next attack)	yes	n.s.
Chaves et al. (14)	TvFF	20 EM (0 male) 20 HC (0 male)	V1, V3, C3, T1 dermatomes	bil.	headache-free	no	n.s.
Malo-Urriés et al. (23)	TvFF	52 migraine (18 males) 30 HC (13 males)	V1, V2, V3, ear, neck, thenar eminence	bil.	not defined	no	n.s.
Szikszay et al. (25)	TvFF	26 EM (5 males) 26 HC (5 males)	V1, forearm	painful side	inter-ictal (48 h since last attack, 48 h before next attack)	yes	n.s.
Pan et al. (28)	EvFF	28 CM (4 males) 64 EM (12 males) 32 HC (5 males)	V1 and T1 dermatomes	L't	inter-ictal, pre-ictal (within 72 h before next attack), ictal, post-ictal (within 24 h of last attack)	yes	pre-ictal, ictal, post-ictal < inter-ictal
Hsiao et al. (29)	EvFF	30 EM (5 males) 27 HC (6 males)	V1 and T1 dermatomes	L't	inter-ictal (48 h since last attack, 48h before next attack)	yes	V1: HC < EM T1: EM < HC

*TvFF, Traditional von Frey filament; EvFF, Electronic von Frey filament; CM, chronic migraine; EM, episodic migraine; HC, healthy control; TM-MOH, transformed migraine with medication overused headache; bil., bilateral; MPT: mechanical punctate pain threshold; n.s., not significant; V1, 2, 3, first, second, and third branch of trigeminal nerve; C3, third cervical nerve; T1, first thoracic nerve*.

### The Methodology of MPT Measurement

Most studies used traditional von Frey filaments to measure MPT (14, 16–18, 22–27), while just two of the studies used electronic von Frey filament (28, 29). The commonly targeted areas of the included studies can be divided into cephalic and extracephalic areas. At least one of the cephalic areas, that is the dermatomes of the first (V1), second (V2), and third (V3) branch of the trigeminal nerve, were included in all the studies, especially V1 dermatome. There is a wider range of targeted extracephalic areas, for instance, upper trapezius muscle (C5), articular pillar of C2-C3, suboccipital muscles (C3), medial forearm (T1), thenar eminence (C6); among these areas, T1 dermatome was most commonly tested. Most studies examined bilateral dermatomes, and some tested on the migraine or more painful side while the others fix the assessments on one side.

### Mechanical Punctate Pain Thresholds During Interictal Phase Compared to Healthy Controls

Most studies (14, 16–18, 22, 25, 28, 29) were done at least once during the “headache-free” or inter-ictal phase. Only one study (23) did not report the criteria regarding the headache phase of the subjects. Among these eight studies, four studies found no significant differences between EM and HC. Two found lower MPT in EM, while two found higher MPT in EM compared to healthy controls (HCs).

Schwedt et al. (16) compared the differences in both thermal and mechanical pain thresholds among patients with EM (inter-ictal phase), chronic migraine (CM), and HC. Regarding the changes in MPT, they did not find any differences between groups. The EM subjects in this study were assessed at least 48 h after being headache-free. Whether these subjects developed headache within 48 h after the assessment was not followed.

Beese et al. (18) conducted an all-female study to investigate the sensory habituation in EM and HC. Among the assessments, the MPT was also included and they found no significant differences between groups. In this study, the EM were tested at least 48 h after the last attack and patients were excluded from the analyses if they experienced a migraine attack within 48 h of the test.

Chaves et al. (14) investigated whether EM concomitant with temporomandibular disorders affects the pain thresholds. However, since the purpose of this article was to compare the differences in MPT between EM and HC or among different phases in migraine, we focused on the comparison between pure EM and HC groups only. They also found no significant differences in inter-ictal MPT between groups. In this study, they described the patients with EM were “not in crisis” at the time of evaluation without a precise description of the time interval between the last or the next headache.

Szikszay et al. (25) investigated whether EM showed altered pain modulation even during the inter-ictal phase. Regarding the MPT assessment, they found no significant differences between EM and HC. The inter-ictal phase of this study was clearly defined as headache free for at least 48 h and those who reported headache within 48 h after the assessment during the follow-up contact were excluded from the analyses.

In the study of Malo-Urriés et al. (23), they compared the differences among patients with EM, cluster headache, tension-type headache, and HC. There were no significant differences in MPT between all groups; however, based on the data provided, we were unable to conclude whether there were significant differences between EM and HC. Moreover, in this study, there was no related description regarding the phase of the EM.

In the studies of Weissman-Fogel et al. (17) and Zappaterra et al. (22), they both found lower MPT in EM compared to HC. The subjects in Weissman-Fogel's study were assessed at least 7 days after their last migraine and in Zappaterra's study, the subjects were described as “out of crisis.” Both studies did not follow up with their subjects to record their next headache attack.

In the study of Pan et al. (28) and Hsiao et al. (29), they both revealed higher interictal MPT in EM compared to HC. The inter-ictal EM subjects in Pan's study were defined as at least 24 h after remission of the last headache attack and at least 72 h before the next headache attack. On the other hand, only the subjects who were in the absence of acute migraine within 48 h before and after the assessments were included for the analyses in the study of Hsiao et al.

In short, the results of MPT compared between EM and HC were inconclusive. Half of the studies found no significant MPT differences between EM and HC, others found lower (25%) or higher (25%) MPT in EM compared to HC.

### Mechanical Punctate Pain Thresholds Compared Between Phases in Patients With Episodic Migraine

Studies also investigated the change of MPT throughout different phases of migraine. In a study of Burstein et al. (26), they compared the sensory thresholds of 42 patients with migraine during the inter-ictal and ictal phases. The authors found that MPT only decreased in patients presenting allodynic symptoms during the ictal phase compared with the inter-ictal phase. Another study of Burstein et al. (27) found that MPTs were lower during allodynic migraine attacks and presented with poor response to triptans. Both studies measured MPT during the inter-ictal and ictal phases; the inter-ictal phase was defined as 5 to 7 days after the last migraine attack; however, the next attack after the assessment was not monitored.

In the study of Yarnitsky et al. (24), they investigated the pain threshold during the inter-ictal and ictal phases. They found lower MPT during the ictal phase compared to the inter-ictal phase over the cephalic area (periorbital) but not over the extracephalic area (forearm). The interictal phase in this study was defined as migraine-free and at least 7 days had elapsed since their last migraine attack. The follow-up interview to confirm the next headache was not mentioned in the study. The ictal measurements were done when the subjects suffered from moderate-to-severe (numerical rating scale: > 7/10) migraine attacks without treatment. Pan et al. (28) divided the EM subjects into the four phases of migraine based on the aforementioned criteria. They found lower MPT during ictal and peri-ictal phases (pre-ictal and post-ictal phases), compared to the inter-ictal phase in both cephalic and extracephalic areas.

## Discussion

### The Potential Role of Allodynia

Approximately 80% of patients with migraine experience cutaneous allodynia (26). Allodynia is when a subject perceives stimuli as painful whereas this intensity of the stimuli usually does not provoke pain, for example wearing glasses or combing hair. The occurrence of allodynia can be assessed subjectively using a questionnaire or objectively using QST, specifically the pain thresholds. To define allodynia with QST, Burstein et al. used a cutoff of one standard deviation (SD) below that of mean thresholds in HC. Later, Jakubowski et al. (30) found that the results with the questionnaire were comparable to the results from QST. All of the included studies in this review used QST to identify allodynia. Yet, these studies report the changes or differences in pain thresholds rather than the presence of allodynia. To report the threshold itself also avoids the potential bias of HC selection. However, the results regarding MPT in patients with migraine remained inconclusive. Therefore, we brought forward some latent reasons to be discussed.

### Was the Inter-ictal Phase the Real Inter-ictal Phase?

Among the twelve studies done during the inter-ictal phase, nine of them explicitly stated that the MPT assessment day was at least 24, 48, 72 h, 5 or 7 days since the last headache. Four of them followed up with the subjects regarding their next attack to exclude or define those during their pre-ictal phase. The rest five studies failed to do so. Two other studies described subjects as “headache-free” without checking their last and next attacks; therefore, the “headache-free” period can be either inter-ictal, pre-ictal, or post-ictal phase, making the comparison difficult. In the two studies that reported lower inter-ictal MPT compared to HC, one study did not define the time interval since the last headache and both studies did not follow to ensure the phases were applied correctly. Since the study of Pan et al. (28) showed low MPT during the pre-ictal and the post-ictal phases compared to the inter-ictal phase as well, periods during the absence of headache attacks should not be considered as the inter-ictal phase for comparison. In the four studies that followed the next headache attack, two of them found higher MPT compared to HC and two found no significant differences. This is in line with the clinical experience that some migraine patients claim themselves to be more tolerable to pain in their daily lives compared to others.

### The Discrepancy of MPT in Current Studies

The results of MPT were rather inconclusive compared to other modalities of quantitative sensory testings (QST). The QST was designed to assess the function of nociceptive Aδ fibers and nociceptive C fibers. The slowly increased heat stimuli (1°C/s) selectively activate the C fibers (31), while pinprick or punctate stimuli mainly activate the Aδ fibers. Whether migraine affects Aδ and C fiber equally warrants future studies to clarify. Another possible reason for the discrepancy between MPT and other modalities is the method for the measurement. The HPT, CPT, and MPT are measured in a continuous manner, that is, with the mode or algometers. Continuous measurement is more likely to detect subtle changes. According to the study of Pan et al. (28) using electronic von Frey filament, the MPT difference between EM and HC was around 20 g. The traditional von Frey filaments might miss the tenuous differences due to their intermittent intensity of measurement.

Sex is also a critical factor of pain sensitivity. A previous study (32) showed that males are less sensitive to pain across all age groups. However, this phenomenon is observed in all QST modalities and therefore, should not be considered as a potential cause for the inconclusive results in MPT. Moreover, in most of the studies in migraine, the control group is age-and sex-matched, which downplayed the possibility of age and sex as confounding of pain sensitivities.

### Is There Enough Evidence for MPT Changes Across Different Migraine Phases?

As a well-acknowledged concept, cutaneous sensitivity (including pain thresholds) fluctuates with the migraine phases. Neuroimaging and electrophysiology studies provide clear evidence of dynamic changes throughout migraine phases. Most studies showed that cortical activity increases before headache attacks (12, 13) (i.e., the pre-ictal phase), but decreased or return to normal activity during the inter-ictal phase (9, 18). Despite the importance of dynamic changes in patients with migraine, longitudinal changes to MPT across the four phases of migraines in the same subjects have yet to be evaluated.

Scholten-Peeters et al. (33) longitudinally measured cyclic changes of pressure pain thresholds (using digital algometer) in patients with migraine across the four phases. The authors found that localized and widespread mechanical sensitivity was more pronounced during the ictal and peri-ictal phases. The study showed that mechanical pain thresholds (indicated by pressure pain thresholds) fluctuate with different migraine phases, in addition to previously reported thermal pain thresholds (8, 16). However, evidence of dynamic changes to MPT measured employing a longitudinal design is required.

## Conclusion and Future Directions

Compared to other modalities of QST, studies of MPT were rather inconclusive. One of the possible reasons may be due to the fundamental differences in the measurement. Most studies used traditional von Frey filaments, which is a nominal measurement unlike the measurement of thermal or pressure pain thresholds. It is easier to detect subtle differences with continuous measurements. On the other hand, the phase of the subjects should be carefully defined with follow-ups to assure the migraine phase was defined correctly. In future studies, the type of measurement and the well-defined migraine phase should be taken into consideration to clarify the MPT differences in patients with migraine. As a cyclic disease, elucidating how MPT changes across different migraine phases is expected to provide new insights into migraine pathophysiology.

## Data Availability Statement

The original contributions presented in the study are included in the article/supplementary material, further inquiries can be directed to the corresponding author/s.

## Author Contributions

L-LP was responsible for the manuscript drafting. L-LP, R-DT, and S-JW contributed to critical revision of the manuscript for important intellectual content. All authors provided the final approval of the version to be published.

## Funding

This work was supported by the Ministry of Science and Technology of Taiwan (MOST 108-2314-B-010-023-MY3 and 110-2321-B-010-005) and by the Brain Research Center, National Yang Ming Chiao Tung University, from the Featured Areas Research Center Program within the framework of the Higher Education Sprout Project by the Ministry of Education of Taiwan.

## Conflict of Interest

The authors declare that the research was conducted in the absence of any commercial or financial relationships that could be construed as a potential conflict of interest.

## Publisher's Note

All claims expressed in this article are solely those of the authors and do not necessarily represent those of their affiliated organizations, or those of the publisher, the editors and the reviewers. Any product that may be evaluated in this article, or claim that may be made by its manufacturer, is not guaranteed or endorsed by the publisher.
